# RETRACTION: Impact of Type 2 Diabetes on Surgical Site Infections and Prognosis Post Orthopaedic Surgery: A Systematic Review and Meta‐Analysis

**DOI:** 10.1111/iwj.70429

**Published:** 2025-03-26

## Abstract

**RETRACTION:** HeC. , ZhouF. , ZhouF. , WangJ. , and HuangW. , “Impact of Type 2 Diabetes on Surgical Site Infections and Prognosis Post Orthopaedic Surgery: A Systematic Review and Meta‐Analysis,” International Wound Journal 21, no. 2 (2024): e14422, 10.1111/iwj.14422.PMC1082872337775974

The above article, published online on 29 September 2023, in Wiley Online Library (http://onlinelibrary.wiley.com/), has been retracted by agreement between the journal Editor in Chief, Professor Keith Harding; and John Wiley & Sons Ltd. Following an investigation by the publisher, all parties have concluded that this article was accepted solely on the basis of a compromised peer review process. The editors have therefore decided to retract the article. The authors did not respond to our notice regarding the retraction.

**RETRACTION:** C. He , F. Zhou , F. Zhou , J. Wang , and W. Huang , “Impact of Type 2 Diabetes on Surgical Site Infections and Prognosis Post Orthopaedic Surgery: A Systematic Review and Meta‐Analysis,” International Wound Journal 21, no. 2 (2024): e14422, 10.1111/iwj.14422.PMC1082872337775974

The above article, published online on 29 September 2023, in Wiley Online Library (http://onlinelibrary.wiley.com/), has been retracted by agreement between the journal Editor in Chief, Professor Keith Harding; and John Wiley & Sons Ltd. Following an investigation by the publisher, all parties have concluded that this article was accepted solely on the basis of a compromised peer review process. The editors have therefore decided to retract the article. The authors did not respond to our notice regarding the retraction.

